# Many Faces of Next-Generation Sequencing in Gene Expression Studies

**DOI:** 10.3390/ijms24044075

**Published:** 2023-02-17

**Authors:** Alexey A. Malygin

**Affiliations:** Institute of Chemical Biology and Fundamental Medicine, Siberian Branch of the Russian Academy of Sciences, 630090 Novosibirsk, Russia; malygin@niboch.nsc.ru

Gene expression is a fundamental cellular process that ensures the transfer of information encoded in a gene into the final functional product. The expression of protein-coding genes is carried out in accordance with the central dogma of molecular biology from DNA by the transcription of genetic information from an active gene to messenger RNA (mRNA) and its subsequent translation. In the process of translation, ribosomes read information in accordance with the genetic code from mRNA and synthesize the corresponding polypeptide. The cell is able to regulate the expression of genes at any stage, since it is vital for maintaining a normal cellular function and allowing it to adapt to environmental changes. Information on the state of the cell transcriptome, which is a complete set of synthesized RNA transcripts, and about its change in response to various events enables the researcher to draw conclusions about the activity of a particular gene and the direction of changes in cellular pathways. However, various cellular mRNAs are differently translated by ribosomes, and sometimes the amount of a newly synthesized protein is not proportional to the amount of its mRNA in the cell. Therefore, it is often important to know the landscape of cellular translatome, i.e., the set of mRNAs translated by ribosomes at a particular moment, from which information about the level of synthesized proteins can be obtained.

Next-generation sequencing (NGS) has provided a huge boost to the studies on gene expression, as evidenced by the explosive growth in the number of papers on this subject deposited in the PubMed over the last years. That is why we have paid particular attention to works utilizing NGS-based methods in the Special Issue of *IJMS* entitled “Regulation of gene expression in the NGS era”. NGS is a revolutionary technology that, when applied to total cellular RNA sequencing (RNA-seq), allows for the profiling of RNA in tissues, cell cultures, and even single cells. This technology makes it possible to obtain a complete portrait of the cell transcriptome, to determine the activity of genes by their expression level, and thus to identify genes that are expressed differently depending on the state of the cells. Similarly, by sequencing mRNAs present in polysomes, i.e., currently translated in the cell, it is possible to determine the profile of the cellular translatome and its detailed characteristics. As a result, the researcher can gain a clear understanding of the gene expression and its regulation at various levels in the cellular systems of interest and under various conditions. An informative presentation of the technical principles underlying NGS, the development of this technology and its further prospects, is briefly provided in the review by Ganguly et al. [1] included in the Special Issue of *IJMS* mentioned above. In this review, the authors provide an excellent overview of recent works in the field of bone marrow aging, focusing in particular on the possibilities offered by NGS-based approaches for this kind research. They fairly argue that NGS paved the way for scientists to explore the transcriptomic profiles of cells residing in the bone marrow in an attempt to understand the molecular mechanisms underlying their aging. It is noteworthy that in this review, the authors also pay attention to describing the future perspectives of NGS-based approaches in aging research, as well as their limitations. Overall, this review clearly demonstrates to the reader the power and value of using NGS-based approaches in aging studies and provides an insight into their potential in other areas of research.

The Special Issue of *IJMS* “Regulation of gene expression in the NGS era”, in addition to the abovementioned review, includes seven research papers that perfectly highlight multiple applications of NGS in studies of diverse aspects of gene expression in various living cells. Two papers in this Issue were performed with bacteria. The first one, by Kotecka et al. [2], was devoted to the identification of the function of the MarR-family transcriptional regulator PA3458 in *Pseudomonas aeruginosa*, a facultative human pathogen with an intrinsic antibiotic resistance mechanism. The authors used RNA-seq to analyze the effect of PA3458 overproduction on the gene expression and revealed changes in the mRNA levels for 133 genes, of which 100 were downregulated, indicating a repressor function of PA3458. This led the authors to the conclusion that PA3458-mediated control is required for *P. aeruginosa* to adapt to high osmolarity. In another paper, Liou et al. [3] applied RNA-seq to compare the expression profiles of *Escherichia coli* grown under aerobic versus microaerobic conditions. They showed that under microaerobiosis, the expression of genes controlling acid stress adaptation, cell adhesion/biofilm formation, electron transport, oligopeptide transport, and anaerobic respiration/fermentation increased. Downregulated genes were involved in iron transport, iron-sulfur cluster assembly, aerobic respiration, and de novo nucleotide synthesis. These findings allowed the authors to identify key changes in the gene expression at the transcriptional level that support *E. coli* growth in low oxygen environments such as the intestine. 

The main portion of the works in the Special Issue were devoted to studies on eukaryotic cell models. Thus, Vizzini et al. [4] studied the transcriptome of cells of the pharynx, the haemopoietic organ in the urochordate *Ciona robusta*, which is a powerful animal model showing a close phylogenetic relationship with vertebrates. Using RNA-seq, they identified more than 15,000 transcripts and, applying bioinformatics analyses, found that the Tgf-β, Wnt, Hedgehog, and FoxO pathways are involved in the *C. robusta* pharynx tissue homeostasis, as in humans. Three more papers were devoted to the use of NGS in the search for molecular manifestations of various pathologies. Baulina et al. [5] searched for novel biomarkers, which could increase the accuracy of diagnosis of hypertrophic cardiomyopathy (HCM), the most common inherited myocardial disease, and improve the understanding of its phenotype. Performing RNA-seq profiling of circulating miRNAs in the plasma of HCM patients, the authors defined miR-499a-5p as one of the upregulated miRNAs. Two other works relate to the study of the impaired expression of the genes of ribosomal proteins. Ribosomal protein genes are among the most actively transcribed genes, while the assembly of a ribosome requires equimolar amounts of about 80 ribosomal proteins, so any disorder of the expression of at least one of these genes leads to a serious imbalance in cellular homeostasis. So, in the first of these works, Gopanenko et al. [6] showed how a deficiency of ribosomal protein eL38, which in the case of mice, leads to the tail-short mutant phenotype being characterized by defects in the development of the axial skeleton, which altered the translatome of human cells. Using the ribosome profiling assay, they revealed many dozens of genes with an increased or decreased translation efficiency, and among the down-regulated genes, there were genes associated with the regulation of transcription and, in particular, with the activation of *Hox* genes responsible for the processes of development and differentiation. In another study, Tian et al. [7], based on the known facts that mutations in the gene *RPS20* encoding ribosomal protein uS10 are often found in patients with a hereditary predisposition to colorectal cancer (CRC), constructed plasmids carrying authentic mutations in the minigene of this protein and transfected human cells with them. By analyzing the transcriptome of cells producing the wild-type of uS10 and its aberrant forms, they found that among the limited number of activated genes, there were a number of genes associated with the progression of CRC, such as *PPM1D* and *PIGN*. Finally, the paper by Postnikova et al. [8], devoted to the study of the features of the expression of the SARS-CoV-2 genome, was also included in this Special Issue. Additionally, although the authors did not use NGS in their study, their finding of a novel ribosome pausing site in the S-protein RNA is extremely important for understanding the mechanism of viral genome RNA expression in infected cells.

In conclusion, all articles published in this Special Issue of *IJMS* clearly illustrate the great potential of NGS-based methods in the study of gene expression and make significant contributions to their own scientific fields. As the Guest Editor, I express my deep gratitude to all the authors who responded to the invitation and submitted their manuscripts.
